# T Cell Engaging Immunotherapies, Highlighting Chimeric Antigen Receptor (CAR) T Cell Therapy

**DOI:** 10.3390/cancers13236067

**Published:** 2021-12-01

**Authors:** Elien De Bousser, Nico Callewaert, Nele Festjens

**Affiliations:** 1 Vlaams Instituut voor Biotechnologie (VIB)—UGent Center for Medical Biotechnology, Technologiepark—Zwijnaarde 75, 9052 Ghent, Belgium; Elien.Debousser@vib-ugent.be; 2 Department of Biochemistry and Microbiology, Ghent University, Technologiepark—Zwijnaarde 75, 9052 Ghent, Belgium

**Keywords:** chimeric antigen receptor, CAR, T cell, immunotherapy

## Abstract

**Simple Summary:**

The ultimate goal of T cell-engaging immunotherapy is to endorse the activity of a person’s own cytotoxic T cells in the tumor microenvironment, finally destroying cancer cells. Several types of immunotherapy are either approved for use or are under study in clinical trials to determine their effectiveness in treating various types of cancer. Chimeric antigen receptor (CAR) T cell therapy is rapidly emerging in the field and has shown unprecedented success in the treatment of hematological malignancies. It entails the collection of a patient’s T cells to genetically engineer them in the lab to express CARs that target surface antigens on tumors to help identify and eradicate the tumor. However, major issues remain to be solved to enable generalized CART cell therapies in the clinic. Novel approaches to tackle these problems are being developed rapidly and are reviewed in this publication.

**Abstract:**

In the past decade, chimeric antigen receptor (CAR) T cell technology has revolutionized cancer immunotherapy. This strategy uses synthetic CARs to redirect the patient’s own immune cells to recognize specific antigens expressed on the surface of tumor cells. The unprecedented success of anti-CD19 CAR T cell therapy against B cell malignancies has resulted in its approval by the US Food and Drug Administration (FDA) in 2017. However, major scientific challenges still remain to be addressed for the broad use of CAR T cell therapy. These include severe toxicities, limited efficacy against solid tumors, and immune suppression in the hostile tumor microenvironment. Furthermore, CAR T cell therapy is a personalized medicine of which the production is time- and resource-intensive, which makes it very expensive. All these factors drive new innovations to engineer more powerful CAR T cells with improved antitumor activity, which are reviewed in this manuscript.

## 1. Introduction

**Figure 1:**
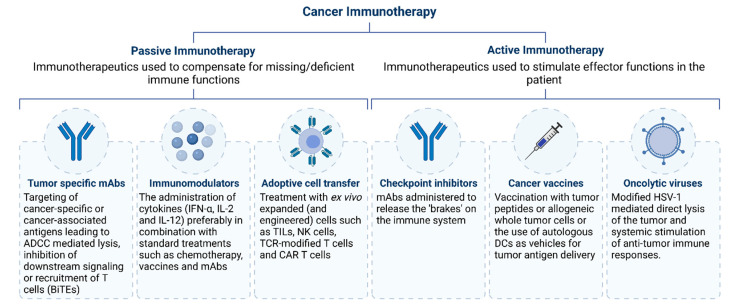
Overview of cancer immunotherapy approaches: ADCC: antibody-dependent cellular cytotoxicity; BiTE: bispecific T cell engager; mAbs: monoclonal antibodies; TILs: tumor-infiltrating lymphocytes; HSV-1: Herpes simplex virus-1.

In addition to routinely applied procedures such as surgery, radiotherapy, and chemotherapy, immunotherapy is becoming a standard approach for cancer treatment. The most frequently used cancer immunotherapy approaches are summarized in Figure 1 and were recently reviewed [1,2]. Cancer immunotherapy aims to promote the activity of cytotoxic T cells within the tumor microenvironment by assisting in the priming of cytotoxic T cells in lymphoid organs and by establishing an efficient and durable antitumor immune response. Generally, three types of T cell-engaging immunotherapies are currently applied in the clinic: immune checkpoint inhibitors (ICI), bispecific T cell engagers (BiTEs), and genetically modified T cells, especially those expressing a chimeric antigen receptor (CAR). In this review, we briefly introduce ICI and BiTEs and mainly focus on the CAR T cell technology, which has lately innovated cancer immunotherapy. The latter strategy uses synthetic CARs to more specifically target cancer cells. Impressive progress has been made in the treatment of hematological malignancies, translated in the FDA approval of several CD19 CAR T therapies [3,4,5,6]. However, considerable challenges still remain for use of CAR T cell therapy to treat solid tumors, preventing them of being approved for use in the clinic. This is mainly due to the hostile tumor microenvironment and CAR-related toxicities, and many approaches have already been explored to overcome these obstacles, on which we further elaborate in the text.

## 2. Immune Checkpoint Inhibitors and Bispecific T Cell Engager Therapy

In 2018, **immune checkpoint inhibitors** (ICI) led to the Nobel Prize in Physiology and Medicine being awarded to Drs. James Allison and Tasuku Honjo, who discovered PD-1 and CTLA-4. Immune checkpoint inhibitors are monoclonal antibodies (mAbs) designed to block co-inhibitory receptors/ligands that induce T cell dysfunction such as PD-1, PD-L1, and CTLA-4. As such, they do not kill tumor cells directly but instead promote priming and activation of pre-existing cytotoxic T cells in the tumor microenvironment (TME). In this way, a durable immune response can be induced [7].

In 2011, the FDA approved the first ICI, Ipilimumab, for patients with unresectable or metastatic melanoma. Ipilimumab is a CTLA-4-targeting mAb and administration of this agent led to a significant increase in overall survival [8]. Anti-CTLA-4 therapy abrogates the CTLA-4-mediated suppression of T cell priming in lymphoid tissues. Since CTLA-4 is also constitutively expressed on Treg cells, anti-CTLA-4 antibodies can inhibit Treg cell functioning too. Hence, antitumor immunity is enhanced by eliminating Treg cell inhibitory functions and increasing the density of cytotoxic T cells in the tumor [9].

Following Ipilimumab, six anti-PD-1 (Nivolumab, Pembrolizumab, and Cemiplimab) or anti-PD-L1 (Atzolizumab, Avelumab, Durvalumab) antibodies have been granted FDA approval for the treatment of multiple cancer types, including melanoma, renal cell carcinoma, hepatocellular carcinoma, and Hodgkin’s disease [10,11].

The response rate of patients to monotherapies blocking PD-1 and CTLA-4 remains modest (only 10–30%) due to emerging mechanisms of acquired resistance to anti-PD-1/PD-L1 or anti-CTLA-4 treatment [12]. Anti-PD-1/PD-L1 therapy in cancer patients leads to the upregulation of TIM-3 expression on Treg cells. TIM-3 further enhances the PD-1 expression level in a feedback loop ultimately resulting in immune resistance and radiotherapy resistance in cancer cells due to the resurgence of Tregs [13].

Combinations of agents targeting these immune checkpoints are now being investigated to increase the survival rates of patients [14]. Examples are the combination of Ipilimumab (anti-CTLA-4) and Nivolumab (anti-PD-1), showing significantly enhanced efficacy in metastatic melanoma patients [15,16] or the inhibition of both TIM-3 and PD-1, preventing the feedback loop of cancer resistance and progression [17].

With more agents under investigation and new immune targets such as checkpoints (BTLA, VISTA, LAG3, and CD47) and co-stimulatory molecules (CD137, OX40 and GITR), the potential for immunotherapy in cancer is broadening.

Another strategy to increase the on-target efficacy of immune checkpoint inhibitor therapy involves the modification of the pH sensitivity of the antibodies. Recently, Zhang and colleagues designed an anti-CTLA-4 antibody by modifying its pH sensitivity to match the acidic pH in the TME [18].

Important to consider is that gains in efficacy must be balanced against a higher frequency and severity of adverse drug reactions. In this sense, novel strategies to promote the immune system through its direct stimulation are under investigation to provide additional clinical improvements of cancer therapy. The costimulatory molecule OX40 is one of the next generation immune therapeutic agents that potentiates the immune system [19].

Although a growing number of several cancer types can efficiently be treated by immune checkpoint inhibitors, the latter only induce remission on the long-term in a minority of patients suffering from only a small set of cancer types, most notably melanoma [20]. A lack of neo-antigenic mutations in combination with defects in either MHC expression or other components of the antigen presenting gear of the tumor cells might explain the intrinsic resistance to immune checkpoint inhibitor therapy. Low numbers of tumor infiltrating T cells combined with the presence of multiple immunosuppressive elements in the TME additionally impair the efficacy of this therapy. Furthermore, the application of ICI in cancer therapy is restricted because of the appearance of autoimmune-like ‘Immune Related Adverse Effects’ (irAEs) caused by uncontrolled immune responses by breaking self-tolerance. The goal is thus to ameliorate immune checkpoint inhibitor therapy by circumventing the initiation of autoimmunity.

The efficacy of ICI is completely or at least partially dependent on inherent, MHC-dependent T cell responses directed against variable and typically undefined tumor-associated antigens (TAAs) or tumor-specific neoantigens. The advantage of this is that the particular neo-antigen needs not to be known, and, in principle, any MHC class I-presented neo-antigen suffices. In addition, T cell-engaging therapies including bispecific antibodies and genetically engineered T cells have been made to readdress cytotoxic T cell specificity toward predefined tumor targets in an MHC-independent way.

**Bispecific T cell redirecting engagers** (BiTEs) are bispecific antibodies that are designed to target CD3 and tumor-specific antigens simultaneously, thereby promoting the cytotoxicity of the patients’ own T cells. In comparison to CAR T therapy, this approach is independent of genetic modification of T cells or the need for ex vivo expansion and manipulation, thereby providing off-the-shelf, immediate therapy [21].

**Figure 2:**
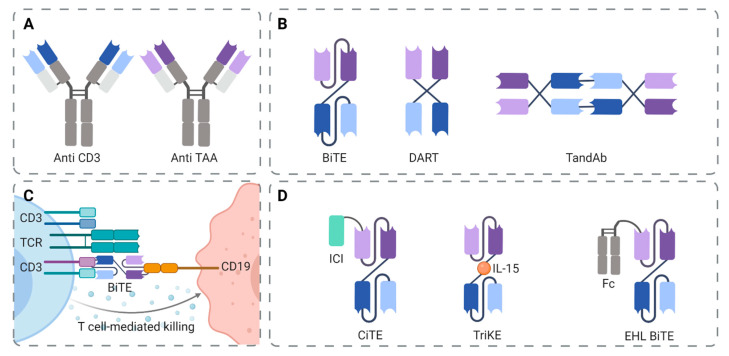
Structure and function of BiTEs: (**A**) Structure of antibodies from which scFv-based BiTEs are derived. (**B**) Structures of bispecific T cell recruiting antibodies. (**C**) MHC-independent targeting of TAAs through the use of BiTE to activate T cells. Example of Blinatumomab is given targeting CD3 and CD19. (**D**) Structural adaptations made to BiTEs to circumvent immune evasion and to optimize stability and half-life. TAA: tumor-associated antigen; BiTE: bispecific T cell engager; DART: dual affinity retargeting antibodies; TandAB: Tandem diabodies; TCR: T cell receptor; ICI: immune checkpoint inhibitor; CiTE: Checkpoint inhibitor T cell engager; TriKE: Tri-specific killer engager; EHL: extended half-life.

Structurally, BiTEs are composed of two single-chain variable fragment (scFv) binding domains derived from monoclonal antibodies, joined by a flexible peptide linker. The first scFv can be designed to target any tumor cell surface antigen. The second scFv is always specific for CD3, the invariant part of the T cell receptor (TCR) complex. Modifications to the structural components of these canonical BiTEs have led to additional therapeutic constructs such as dual-affinity retargeting antibodies (DART) and tandem diabodies (TandAb) (Figure 2A,B) [22]. DARTs consist of a crisscross format design, where the two heavy chain variable fragments have been exchanged with one another, rendering them more stable. In preclinical studies, DARTs have shown enhanced cytotoxicity in comparison to BiTEs [23]. TandAbs contain two single-chain diabodies, with four variable domains providing dual antigen-binding sites, and are joined by disulfide bonds, thereby enhancing their stability and optimal interaction between target and effector cell [24].

BiTEs induce effective T cell responses at very low concentrations of 10 to 100 pg/mL and at very low effector cells to target cell ratios (<1/90) [25]. The small size, high flexibility, and high affinity interaction between effector and target cells are the key features underlying the high efficacy of BiTEs [26].

When a BiTE engages both the tumor cell and the cytotoxic T cell, T cell proliferation and differentiation are induced, ultimately leading to cytotoxic T cell effector functions and tumor cell killing. In this way, T cells can perform serial-target lysis in the presence of BiTEs by rapidly binding and killing many cells [27] (Figure 2C). BiTE therapy poses a strategy to reactivate T cells that become exhausted through long-term exposure to tumor antigens. This is because BiTEs mediate target cell killing mainly via (non-tumor) antigen-experienced T cells that differentiate to effector memory T cells after activation and these cells can be reactivated without costimulatory signals such as CD28 and IL-2 [28]. Furthermore, the immunological synapse formed between T cells and tumor cells is essential to effective BiTE-mediated tumor lysis. As BiTEs have only one CD3-specific binding domain, bispecific antibodies can monovalently bind to all T cells but with rather low affinity. Consequently, T cell signaling through CD3 is only triggered when the BiTE is presented to the cell in a multivalent fashion by the target cell [29].

Blinatumomab (Blincyto^®^ by Amgen) was the first CD19 targeting BiTE approved by the FDA for the treatment of B cell malignancies such as acute lymphoblastic leukemia (ALL) and for the treatment of patients experiencing minimal residual disease (MRD) [30]. Its efficiency in the treatment of acute myeloid leukemia (AML) and non-Hodgkin lymphoma (NHL) is currently being evaluated in clinical trials. Although Blinatumomab is a promising drug to treat ALL, this antibody is still limited in clinical practice. AFM11 (by Affimed), a new TandAb with a higher affinity to both CD3 and CD19, a higher efficacy, and a longer half-life was designed and shows promising results in preclinical trials [24]. Since the FDA approval of Blinatumomab, BiTEs for the management of hematologic malignancies are being developed rapidly.

In addition to CD19, BiTEs were developed that target differentiation antigens of which the expression profile is restricted to specific hematopoietic cell lineages and their respective malignant counterparts. Examples include B cell maturation antigen (BCMA) expressed by plasma cells and multiple myeloma, and expression of CD33 by AML and myeloid cells [31,32]. Several of these novel BiTEs are currently being tested in clinical trials and were recently reviewed by Tian and colleagues [33].

Beyond hematological cancers, a multitude of BiTEs targeting a diverse range of TAAs present in solid cancers are currently under clinical investigation. However, the selection of appropriate target antigens is crucial to avoid on-target, off-tumor effects. Based on the observation that prostate-specific membrane antigen (PSMA) is restrictedly expressed in both malignant and non-malignant prostate tissue, the canonical BiTE Pasotuximab was designed. This is the first example showing that BiTE therapies can be effective in the treatment of solid tumors [34]. Other examples of BiTEs for solid tumor in clinical trials include BiTEs targeting EGFRvIII for treatment of glioblastoma, Delta-like protein 3 targeting for small cell-lung cancer, and Mucin 17 targeting for gastric cancer [35,36].

Although BiTEs are shown to be efficient in many hematological malignancies, a subset of patients still show limited or no response to BiTEs. The suppression of T cells in the TME is an important reason for this tumor escape. Multiple (pre)clinical trials are therefore focusing on the combination of BiTE therapy with ICI, such as PD-1/PD-L1 inhibitors [37]. Furthermore, significant progress has been made in the design and development of bifunctional checkpoint-inhibitory T cell engagers (CiTEs) in order to tackle immune escape (Figure 2D). In this molecule, locally restricted ICI action is combined with the T cell redirecting function of the BiTE, in order to enhance the effectiveness of the BiTE part of the molecule while limiting the systemic activation of T cells due to adverse effects linked to ICI [38].

Loss of target antigen expression is another mechanism underlying tumor escape and can be caused by antigenic shift and disrupted target antigen trafficking [39]. This observation led to the development of combinatorial, multi-targeting immunotherapies in order to reduce the incidence of immune escape and disease relapse. In this regard, simultaneous multiple interaction T cell engagers (SMITEs) were designed that are composed of two independent BiTEs to enable concurrent recognition of two different TAAs on the tumor cell and of CD3 and CD28 on the effector T cells [40]. As such, they provide T cell co-stimulatory signals and can overcome antigen escape or heterogeneity.

Additionally, the new generation of modified antibodies with tri-specificity, also known as tri-specific killer engagers (TriKEs), are predominantly designed to target the activity of antitumor T cells or NK cells, and consist of a bispecific antibody linked to an immunostimulatory cytokine domain in order to augment host immune function (Figure 2D). For example, an CD33/CD16/IL-15 TriKE redirects NK cells toward myeloid blasts and is under clinical investigation in AML patients [41].

Due to the low molecular weight of scFv-based BiTEs, their half-life is short (for example ±2 h for Blinatumomab). Therefore, BiTEs have to be administered by continuous or repeated intravenous infusion to maintain their therapeutic concentration [42]. This is now being improved by the development of ‘extended half-life’ (EHL) products by modifying BiTE molecules with Fc domains (Figure 2D). Novel IgG-based T cell bispecific antibodies typically use a bispecific IgG backbone and Fc regions with reduced or abolished binding to the Fcγ receptor. For example, a bispecific CD20/CD3 T cell dependent antibody was developed by Genentech [43], and Macrogenics fused a Fc region to a bispecific diabody-based CD19/CD3 DART [44]. As such, the half-life can be increased to over 200 h after single dosing. Furthermore, solubility and stability are improved by the inclusion of these Fc domains [33]. Fc domains can also recruit NK cells and macrophages to induce antibody-dependent, cell-mediated cytotoxicity (ADCC) and complement-dependent Cytotoxicity [45]. However, this may also lead to an increase in adverse effects such as cytokine release syndrome (CRS) and neurological adverse events that are often related to immunotherapies, which are discussed in more detail in the context of CAR T cell therapy.

## 3. Adoptive T Cell Transfer Therapy—A Focus on CAR T Cells

Although targeted therapy and immunotherapy with ICI have greatly improved the survival of melanoma and NSCLC patients, a large proportion of patients still develop disease progression upon receiving these therapies [46]. Adoptive cell therapy (ACT) may provide an additional treatment option for these patients. ACT is a cell-based immunotherapy that utilizes the patient’s own immune cells (autologous transfer) or immune cells from a donor (allogeneic transfer) in order to find and eliminate tumor cells. Immune cells can be adapted and expanded ex vivo before they are transferred back into the patient as a therapeutic product. For the treatment of cancer, ACT-based strategies have been set up to enhance T cell-mediated antitumor immunity while at the same time limiting the immune suppressive mechanisms in the TME. Hence, ACT currently represents the most rapidly expanding modality of modern cancer immunotherapy and comprises over 30% of the entire immune–oncology pipeline [47].

ACT products mainly consist of tumor-infiltrating lymphocytes (TILs), TCR-modified T cells, and CAR-modified T cells, and are the main focus of this section (Table 1). The use of other immune cells such as DCs and NK cells as a basis for cell therapy is also under investigation and is shortly introduced.

### 3.1. Tumor Infiltrating Lymphocytes

The presence of TIL in tumor tissue is an indication of an antitumor immune response by the host and correlates with favorable clinical outcome in several tumor types, mostly melanoma [49]. In the TIL treatment protocol, patients undergo the resection of one or more metastases. The resected tumor is then dissociated enzymatically and cultured in medium containing IL-2 for a period of two weeks, resulting in the proliferation of TILs. At least 50 × 10^6^ TILs must be obtained in this step to be able to expand further in the presence of stimulation and cytokines. During this 2-week expansion phase, up to 10^11^ cells are obtained that are harvested and prepared for infusion into the patient [50].

Prior to infusion, patients have to undergo lymphodepletion. A lymphodepletion regimen generally consists of infusion of cyclophosphamide (60 mg/kg) and fludarabine (25 mg/m^2^) and enhances both the rate and the duration of clinical responses [51]. Furthermore, after the infusion of TILs, patients receive a high dose of IL-2, and subsequent daily support with IL-2 is thought to further enhance the survival and clinical efficacy of TILs [52]. The exact cellular and molecular mechanisms underlying the enhanced function of TILs after lymphodepletion are still under investigation. Lymphodepletion increases the availability of IL-7 and IL-15 in the absence of endogenous lymphocytes, thereby enhancing the T cell homeostasis end expansion of transferred therapeutic cells [53]. Furthermore, lymphodepletion can enhance the efficacy of TILs through the eradication of immunesuppressive cells such as MDSCs and Tregs [54].

Only about 30% of TILs are tumor reactive. Moreover, tumors with low levels of unique TAAs are likely to contain limited numbers of TILs, and can therefore give rise to TIL ACT products with inferior efficiency. Thus, selecting for tumor reactive TILs can significantly reduce both the culture time and the number of cells that need to be infused. The identification and enrichment of TILs with TCRs specifically recognizing tumor neoantigens is probably the most advanced approach to enable personalized TIL therapy. A possible strategy, designed by Rosenberg et al., encompasses comparative analysis of the next generation sequencing (NGS) exome sequencing data of healthy tissue and tumor for the identification of mutated proteins. Using an MHC-binding algorithm, presumed T cell epitopes are subsequently identified, synthesized, and appraised for recognition by TILs [55]. The neoantigen-specific T cells are then purified using flow cytometry and expanded ex vivo. This strategy has led to favorable outcomes in patients with metastatic cholangiocarcinoma, colorectal cancer, and breast cancer [56].

The composition and T cell phenotype of TILs play an important role in determining the therapeutic outcome after ACT. For example, PD-1 expression was observed to be high on melanoma reactive TILs. As this population can recover function after culturing in the presence of IL-2, their presence is associated with an enhanced tumor reactivity [57]. Furthermore, the selection of tumor-reactive T cells based on the high expression of co-stimulatory molecules such as 4-1BB or OX-40 may contribute to a ‘fit’ T cell product, as it is expected that these cells are less prone to lose antitumor effector functions during expansion in IL-2 culture conditions [58]. Moreover, T_RM_ cells which are characterized by increased homing capacity to the tumor were associated with better survival outcomes in many solid tumors, and T_SCM_ cells are favored because of their ability to self-renew [59,60]. As both CD8^+^ and CD4^+^ T cells play an important role in efficient antitumor immunity, the inclusion of both T cell subsets may be a good therapeutic strategy to generate effective TIL ACT products [61].

Based on reported data on TIL ACT studies, the most efficient tumor rejection upon TIL treatment is seen in melanoma patients. An average objective response rate for TIL ACT in the presence of high IL-2 of 44% is reached over multiple studies [62]. This is comparable to anti-PD-1 ICI therapy but lower than that with combined anti-PD-1 and anti-CTLA-4 treatment. Up to now, no FDA approval is received for TIL ACT for the treatment of solid tumors, however this strategy is in active clinical development, and multiple clinical trials have already reported promising results. For example, IOVANCE Biotherapeutics developed Lifileucel, a commercial autologous TIL product that is currently being tested in a phase II clinical trial for patients with unresectable or metastatic melanoma, HNSCC and NSCLC. An overall response rate of 36.4% was achieved after a single infusion regimen combined with lymphodepletion and IL-2 administration [63]. In comparison, chemotherapy, which is the only available treatment option for these patients to date, is effective in only 10% of the patients and responses are typically transient.

In the context of solid tumor immunotherapy, in particular melanoma, ICI agents are still dominating, and there are challenges associated with TIL ACT, making them less favorable than ICI. For example, as ICI therapies are universally applicable and ubiquitously available, patients can be treated as soon as it is needed. TIL-based therapy on the contrary, need to be custom-made for each individual patient through a process requiring specialized GMP facilities. IOVANCE Biotherapeutics reported the shortest TIL production process (22 days), but other groups report a longer production time of at least 6–8 weeks [64]. Moreover, TIL-based therapy is considerably more expensive than ICI. Taking all of the above into account, it would be advisable to use TIL ACT as a second-line therapy in patients who showed to be unresponsive or have an acquired resistance to ICI, or in patients with an unfavorable expression profile of ICI response biomarkers (for example, PD-L1 immunohistochemical staining) [65].

### 3.2. TCR-Modified T Cells

As is clear from the previous section, adoptive transfer of antigen-specific T cells is a desirable approach for cancer immunotherapy. However, obtaining sufficiently high numbers of T cells by selection and expansion procedures is challenging, as these cells may be low in numbers or in an exhausted state due to the hostile TME. Using engineered T cells can overcome these difficulties. In this approach, the patient’s T cells are reinforced to obtain antitumor reactivity by viral transduction of tumor-specific antigen-targeting receptors. As TCRs recognize internal antigens, TCR-engineered T cells have a broad scope of possible targets. Another major benefit of TCRs is the low level of presented antigen needed for their activation. Due to this high sensitivity, cancer neoantigens can be targeted that arise from somatic mutations, resulting in mutant proteins that are only present in the tumor, and not in normal tissue [66].

In TCR gene therapy, autologous T cells are redirected to recognize tumor antigens in an MHC-dependent manner by introduction of genes encoding TCR-α and β chains. Neoantigens and the TCRs that specifically recognize them can be identified through sequencing of the patient’s tumor cells in order to identify tumor-specific mutations. These potential antigens are then further processed through an MHC-binding prediction algorithm to enrich the antigens that are most likely to be presented to T cells. After further validation using human leukocyte antigen (HLA)-tetramers, TCRs are identified that target these antigens through clonal T cell analysis or single cell sequencing. Strategies to identify and characterize tumor antigen specific TCRs were recently reviewed [67].

Upon successful identification of the individual tumor-reactive TCR chains, the corresponding genes need to be safely and efficiently transferred to reconstitute the functional TCR. In clinical practice, peripheral blood T cells are obtained from the patient through leukapheresis and are subsequently engineered by gamma-retroviral or lentiviral vector transduction in order to incorporate the TCR genes into the host genome, resulting in the high-level expression of the transgene TCR. Alternatively, genetic engineering methods based on the Sleeping Beauty-derived transposon/transposase system or CRISPR/Cas9-based technologies are currently under development [68,69].

As described above for ACT with TIL, most treatment regimens for TCR-modified T cells include the preconditioning of the patient through lymphodepletion in order to facilitate the engraftment and persistence of the modified T cells after infusion. Beyond lymphodepletion, multiple studies aim to reduce the number of endogenous T cells through total body irradiation prior to ACT. Furthermore, the administration of IL-2 following T cell infusion is often included in the treatment protocol [56]. However, both lymphodepletion and systemic IL-2 administration can cause severe toxicity in the patient, and this motivates them to genetically engineer TCR-modified T cells to enhance their survival and persistence in vivo, as is described further.

Current ACTs mainly focus on the CD8^+^ T cell subset as cytotoxic effectors. However, recent evidence has shown that CD4^+^ T cells also exert antitumor efficacy, and that these cells have also been part of the TCR infusion product used in early clinical trials [70]. However, tumors only express antigens expressed on MHC class I molecules which na-tural CD4^+^ T cells cannot recognize. An emerging solution aims to co-transfer the CD8 coreceptor together with the MHC class I-restricted TCR and the CD4 coreceptor together with the class II-restricted TCR in order to achieve optimal TCR function in all engineered T cells [71]. Alternatively, the affinity of the TCR toward pMHC could be enhanced in order to compensate for a reduction in avidity, thereby eliminating the need for signaling through the CD4 or CD8 coreceptor. However, naturally occurring high-affinity TCRs are in the minority because of central and peripheral tolerance mechanisms. Therefore, improved affinity of the TCR for the cognate peptide/MHC complex could be obtained through the introduction of mutations into the CDR regions of the TCR in order to meet antibody-like affinities that are usually 1000-fold higher. However, after affinity maturation, the specificity of the TCR may be reduced, which could lead to cross-reactivity and toxicity [72].

As TCR-modified T cells express the introduced TCR along with the endogenous TCR, this may result in mispairing between the endogenous and introduced TCR chains, thereby producing unknown specificities and toxicity [73]. Furthermore, reduced TCR signaling can occur due to competition between the introduced and endogenous TCR chains for access to CD3 signaling molecules. Efforts to prevent this mispairing involve codon optimization of the transgenes, leading to enhanced functional expression of the transgenic TCR, and the addition of cysteine residues to introduce disulfide bonds between the chains [74]. Furthermore, it is possible to introduce the novel TCR while simultaneously knocking out the endogenous TCR. In a recent clinical trial, T cells were engineered with an NY-ESO-1-targeting TCR in addition to CRISPR knockout of both the endogenous TCR and PD-1 encoding genes [75]. The introduced TCR gene can also be specifically targeted towards the TCR-α and -β locus, resulting in direct TCR gene replacement, allowing physiological expression of the engineered TCR [76].

Moreover, in this regard, efforts are being made in the engineering of γδ T cells and NK cells for ACT. As these cells do not express a polyclonal endogenous αβ TCR repertoire, mispairing is circumvented when a new TCR is introduced, and the risk for allo-reactivity and graft-versus-host disease is reduced. Moreover, these cells have been shown to have some natural anticancer reactivity. However, to turn this kind of therapy in an allogeneic, ‘off the shelf’ cell product, additional engineering is required to eliminate immunegenic antigens including HLA, and to circumvent unwanted immune responses after adoptive transfer into patients [77].

Lipid-restricted CD1 T cells and monomorphic MHC class I-related protein (MR1)-restricted T cells are naturally occurring T cell subsets with an MHC independent αβ TCR. These cells recognize ligands related to tumor or pathogen-derived lipids, metabolites, phospho-antigens or stress-ligands, and are emerging as attractive alternatives for TCR-modified T cell therapy [78]. Recently, an MR1-restricted TCR was identified (designated clone MC.7.G5), with specificity against a wide variety of tumors but not against healthy tissue or pathogens [79]. This observation further gives rise to the opportunity to develop population wide, ‘off the shelf’ TCR-modified T cells, alleviating the need for patient specific MHC restriction.

Genetically engineered TCR T cells could be modified in various ways to fight cancer immune evasion, which is reflected by the very large number of current clinical trials involving ACT. Strategies to increase the potency of ACT involve multiple genetic modifications in order to increase tumor specificity, homing, T cell persistence, and tumor elimination, and are described in more detail in the next paragraphs in the context of CAR T cell therapy, in addition to being recently reviewed in the context of TCR gene therapy [80,81].

TCR-modified T cell therapies have been slow to reach clinical approval, but there have been studies that show the effectiveness of this treatment, leading to over 70 clinical trials [82]. Tumor antigens that are most often targeted include tissue-specific antigens such as melanoma differentiation antigens, cancer/testis antigens, overexpression antigens, neoantigens, and viral antigens.

The first evidence of clinical feasibility and potency was delivered by TCR-modified T cells that target the melanoma differentiation antigen MART-1 in patients with progressive metastatic melanoma [83]. Another promising example is the NY-ESO-1 TCR in the treatment of patients with metastatic synovial sarcoma [84]. Antitumor responses of 50% were reached in these patients who have limited treatment options [85].

Hence, so far, the concept appears to be most successful with protein antigens that are commonly de novo expressed in a substantial fraction of patients suffering from a particular tumor type, the peptides of which are presented by the major HLA class I haplotypes, such that a single TCR can be used to engineer the cells of a good proportion of patients. The ‘personalized’ concept of finding neoantigens and engineering a customized patient-specific TCR is more cumbersome and may remain a niche area in an era where bispecific T cell engagers and CAR T cells are more to the forefront.

### 3.3. Chimeric Antigen Receptor (CAR) T Cells

CAR T cells, initially called T bodies, are T cells expressing a transgenic antigen-specific chimeric receptor that combines antibody specificity with T cell effector and regulatory functions. CAR T cells were first described in the late 1980s by Eshhar and colleagues [86]. This group replaced the antigen recognition variable regions of the TCR chains with the variable regions of an anti-SP6 antibody. While antigen specific T cell activation was demonstrated in this way, multiple technical hurdles had to be tackled to come to the efficient single-chain design, which has been the backbone for the majority of CAR T therapies to date.

#### 3.3.1. Basic Principles of CAR T Design and Production

The **extracellular domain** recognizes the cell surface tumor antigen independent of MHC restriction. The predominant type of extracellular ligand recognition domain in CARs is a single chain variable antibody fragment or scFv derived from tumor antigen-reactive antibodies. Here, the antibody variable light chain is tethered to the variable heavy chain of the same antibody by a peptide linker. An important step in the design of the CAR molecule is the selection of a scFv with optimal affinity to the target antigen. While high affinity may lead to reduced mobility and thus the serial killing capacity of the CAR, a low affinity leads to inappropriate cellular activation of the CAR T cell [87].

Nanobodies, the variable regions of heavy chain only antibodies, can also be used as antigen recognition domain in the design of a CAR. Nanobodies are smaller in size compared to traditional mAbs and have stable physiochemical properties. Of importance is the high solubility/lack of aggregative properties, a notorious problem with the scFv format that can lead to non-specific interactions, obviously to be avoided in the CAR T context [88]. Moreover, due to the high sequence homology with the human VH3 gene family, nanobodies present a relatively low immunogenicity in humans as compared to murine scFvs, and can be largely humanized [89]. The use of nanobodies in CAR design can thus overcome some limitations of scFvs, such as the complex folding and assembly steps and lower protein stability.

Currently, scFvs are most often used as extracellular ligand recognition domain. However, the first chimeric receptors were antibody variable regions (Fab) fused to TCR constant regions [90]. More recently, CD19-specific antibody Fab fragments were fused to TCR γ and δ constant regions to enable the assembly of so-called antibody TCRs, which efficiently associate with the endogenous CD3 signaling complex, resulting in more physiological activation. This strategy also avoids the mispairing with endogenous TCR chains, as was described in the previous section about TCR-modified T cells. In comparison with classical CARs, reduced toxicity and exhaustion was observed [91].

Alternatively, Celyad Oncology is investigating CAR T cells based on NKG2D, a receptor expressed on natural killer cells that binds to multiple stress-induced ligands (NKG2DL) expressed on a broad range of tumor cells [92,93].

The extracellular antigen recognition domain is linked to the **intracellular signaling domains** by transmembrane and spacer domains. As some antigens are localized closer to the plasma membrane (PM) of the tumor cell than others, this spacer needs to be empirically optimized for each application with respect to antigen binding and T cell activation. However, while a longer linker enhances the chance for antigen recognition closer to the tumor cell membrane, it may also elicit an immune response, leading to the deletion of the CAR in vivo [94].

**Figure 3:**
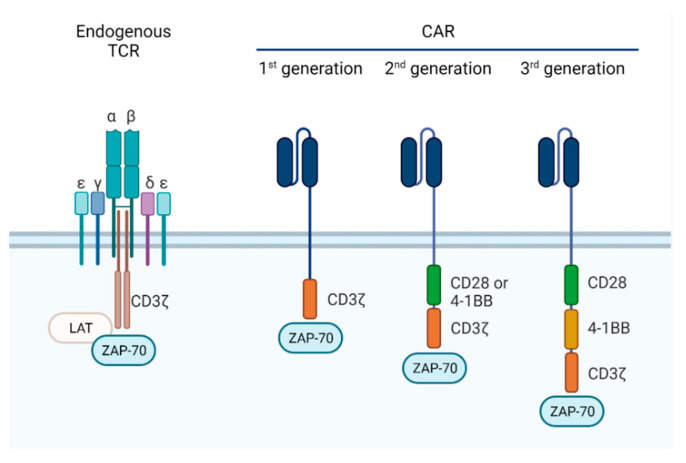
Schematic overview of the CAR design through multiple generations. On the left side of the figure, the endogenous TCR is depicted together with the proximal interacting signaling molecules (LAT: linker of activation and ZAP70). On the right side of the figure, the different successive generations of the CAR design are depicted. CAR domains: blue domain: antigen recognition domain; green domain: costimulatory domain; orange domain: CD3ζ signaling domain.

The intracellular signaling domain of the TCR complex transduces ‘signal 1’ to initiate the signaling cascade. Co-stimulatory receptors, CD28 in particular, convey ‘signal 2’, which is important for sustained signaling, proliferation, and prevention of anergy. CAR T cells are not activated via endogenous TCR signaling moieties but instead via intracellular signal transducing domains that are integrated into the synthetic cell surface receptor. The original, first generation CARs were composed of only the intracellular part of the CD3ζ chain (Figure 3) Alternatively, FcRγ and Syk kinase domains were studied as potential signaling domains [95]. Although these CAR T cells are activated and exhibit cytotoxicity upon antigen recognition, they fail to proliferate and elicit long-term antitumor responses in vivo [96].

Only after the incorporation of additional co-stimulatory receptor domains, CD28 and 4-1BB in particular, the so-called second-generation CARs could provide the efficacy that ultimately led to FDA approval in 2017 for CAR T cell treatment of hematological malignancies [97]. Intracellular signaling domains of costimulatory receptors are usually engineered at the membrane proximal position, followed by the CD3ζ in the distal position (Figure 3) [98]. In the presence of this second activation signal, the CAR T cells show long-lasting cytokine release, proliferation, and antitumor responses, irrespective of the presence of the costimulatory receptor ligand in the TME. In general, different signaling domains induce varying degrees of activation and have a distinct impact on CAR T cell activity, proliferation, and fate. For example, CD28 costimulation increases glucose uptake and ATP generation, which favors an immediate response by effector T cells, while 4-1BB increases catabolism and oxidative phosphorylation, inducing a central memory fate and long-term survival of T cells [99]. Other co-stimulatory domains derived from OX40, ICOS, or CD27 are also being incorporated in CAR design. The different costimulatory receptors, and the implications for CAR functionality, are summarized in Table 2 [100].

More recent optimization has led to the development of third-generation constructs incorporating more than one of the above-mentioned costimulatory domains alongside the CD3ζ chain. When CD28 costimulation is combined with, for example, OX40 or 4-1BB, cytotoxicity and durability is further enhanced, and lower doses of the CAR T cells are required for full antitumor activity. For example, a study comparing CD19 CARs with either CD28 or CD28 and 4-1BB costimulatory domains revealed that third-generation CARs obtain higher signal strengths compared to second-generation CARs [109]. Moreover, studies have indicated superior tumor eradication and increased persistence in vivo when CD28 and 4-1BB domains are combined in a CD19 CAR [110]. However, the added value of the third-generation format is under debate, as some studies have shown that no increase in terms of antitumor potency can be observed [87]. The optimal dose of third-generation CAR T cells leading to improved clinical efficacy but without increased risk for toxicities is yet to be determined.

The modular composition of the CAR has several benefits for use in ACT. Due to the antibody-based targeting, antigen recognition is rendered independent of MHC presentation, a feature often deficient in cancer cells. Furthermore, basically all antigens could be targeted, including non-classical T cell antigens, such as carbohydrates, lipids, or structural variants of antigens, as long as a targeting molecule is available. Of course, this binding molecule should not be immunogenic, to avoid deletion of the therapeutic cells. This aspect of CAR T cell engineering has so far been rather poorly studied. In contrast, CAR T cells are not able to recognize intracellular antigens. Some progress has therefore been made to design CAR T cells with TCR-like antibodies recognizing MHC-bound peptides, for example, for NY-ESO-1 peptide presented by HLA-A2 [111].

The **manufacturing of CAR T cell** **products** requires the adherence to good manufacturing practice (GMP) rules [112]. In short, the procedure includes the collection of immune cells from the patient by leukapheresis, activation in the presence of different stimuli (e.g., anti-CD3 antibodies), the introduction of the CAR transgene by genetic engineering, T cell expansion, and quality control of the final cell product before administration back to the patient. The CAR transgene is delivered to T cells by means of plasmid transfection or viral transduction with vectors based on adenovirus, retrovirus, or lentivirus. Of these methods, lentiviral vectors are most often used to transduce human T cells.

CAR T cells can also be engineered through mRNA electroporation, with the advantage that transient CAR expression leads to reduced toxicity. However, the advantage of persistent antitumor immunity is lost, which is one of the most compelling advantages of ACT versus protein-biopharmaceutical interventions, in particular BiTEs [113].

Alternatively, stable introduction of transgenes can be obtained by using synthetic DNA- or mRNA-transposon systems based on the Sleeping Beauty transposon. In this application, a transposon vector (plasmid or mRNA coding for a mobilizing transposase protein) can be delivered through nucleofection for stable integration into the genome [114]. Importantly, the Sleeping Beauty-based transposon system appears to be less mutagenic as compared to viral vectors, as its genomic integration occurs largely at random, in contrast to retro- and lentiviruses, which have the tendency to integrate in transcriptionally active sites. However, the risk of insertional mutagenesis and subsequent oncogenic transformation is low with the currently used classic transgene delivery systems to engineer mature T cells, and no oncogenic events as a result of T cell transformation have been reported so far.

#### 3.3.2. The Clinical Success of Second-Generation Anti-CD19 CAR T Cells

The first CAR T cell clinical trials initiated about 20 years ago and targeted the folate receptor to treat patients with advanced epithelial ovarian cancer [115]. However, the breakthrough was achieved in the subsequent years with CD19-specific CAR T cells targeting B cell malignancies. For the majority of patients, complete or partial response was reported. For example, CD19 CAR T therapy demonstrated high efficiency in curing ALL. CD19 is almost an ideal target in treating B-ALL for its higher expression on the surface of tumor cells. Kymriah (Tisagenlecleucel), a CD19 CAR with 4-1BB costimulatory domain developed by Novartis (Basel, Switzerland), has been approved by the FDA [3]. Furthermore, Axicabtagene Ciloleucel (Yescarta, Gilead Sciences, California, CA, USA), a CD19 CAR with CD28 costimulatory domain developed by Kite (a GILEAD company, California, CA, USA), was approved to treat relapsed diffuse large-B-cell lymphoma [4]. Moreover, Brexucabtagne Autoleucel (Tecartus, developed by Kite, California, CA, USA), a CD19 CAR with CD28 costimulatory domain, was approved as the first and only CAR T cell product for the treatment of adult patients diagnosed with mantle cell lymphoma [5]. More recently, Lisocabtagene Maraleucel (Breyanzi by Juno therapeutics, Washington, WA, USA), a CD19 CAR with 4-1BB costimulatory domain, was approved for the treatment of adult relapsed or refractory large-B-cell lymphoma [6].

Additionally, CD19 CAR T cell therapy is showing promise in other hematological cancers such as multiple myeloma [116]. This is remarkable because multiple myeloma entirely consists of plasma cells which are CD19 negative. Possibly, the CD19 CAR T cells eliminate a CD19 positive cancer stem cell population or a suppressor B cell population. Furthermore, BCMA and CD128 are studied as other promising targets in multiple myeloma [117]. Very recently, FDA approval was granted for Idecabtagene Vicleucel (Abecma developed by Celgene Corporation, New Jersey, NJ, USA), the first cell-based therapy (an anti-BCMA CAR with 4-1BB costimulatory domain) to treat adult patients with multiple myeloma. Currently, nearly 400 trials using second generation CAR T cells are under clinical investigation, and the progress in CAR T cell therapy for hematological malignancies was recently reviewed elsewhere [118,119].

### 3.4. Breaking Bottlenecks in CAR T Cell Therapy

**Figure 4:**
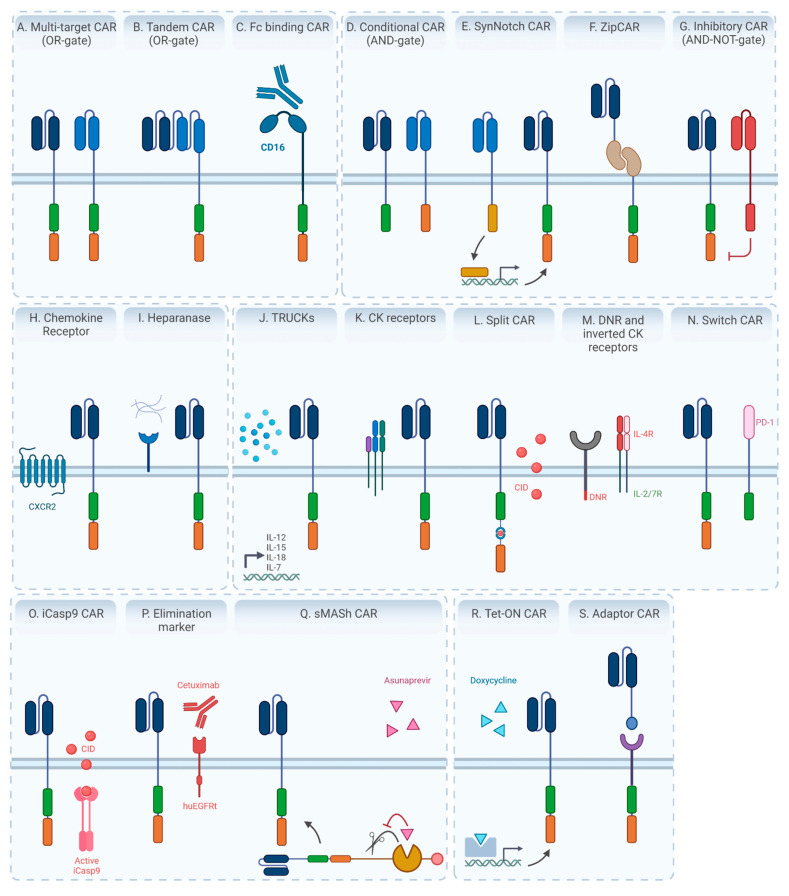
Schematic representation of next-generation CAR T cells described in the text: SynNotch: Synthetic notch receptor; TRUCKs: T cells redirected for antigen-unrestricted cytokine-initiated killing; CK: cytokine; CID: chemical inducer of dimerization; DNR: dominant negative receptor; iCasp9: inducible Caspase 9; huEGFRt: truncated humanized epidermal growth factor receptor; sMASH: small molecule-assisted shutoff; Tet: Tetracycline. CAR domains: blue domain: antigen recognition domain; green domain: costimulatory domain; orange domain: CD3ζ signaling domain. (**A**) Multi-target CAR (OR-gate), (**B**) Tandem-target CAR (OR-gate), (**C**) Fc-binding CAR, (**D**) Conditional-target CAR (AND-gate), (**E**) SynNotch CAR, (**F**) Zip CAR, (**G**) Inhibitory CAR (AND-NOT-gate), (**H**) Chemokine Receptor, (**I**) Heparanase, (**J**) TRUCKs, **(K**) CK receptors, (**L**) Split CAR, (**M**) DNR and inverted CK receptors, (**N**) Switch CAR, (**O**) iCasp9 CAR, (**P**) Elimination marker, (**Q**) sMASh CAR, (**R**) Tet-ON CAR, (**S**) Adaptor CAR.

Although highly encouraging clinical results have spurred massive research efforts into CAR T cell therapy, major scientific challenges still hamper full clinical adoption due to toxicities and challenges associated with the treatment of solid tumors, especially those of epithelial origin (carcinomas). Recent advances in solid tumor CAR T therapy, considering important targeted surface markers in various cancers and their efficacies, are listed in a recent review by Marofi et al. [120]. Response to CAR T therapy is variable due to both heterogeneity in target-antigen expression in malignant cells, as well as the high rates of antigen escape and downregulation of tumor antigens. Furthermore, CAR T cells often lack the ability to persist and maintain their effector function in the TME. In this section, an overview is given on the main bottlenecks in CAR T therapy and how these may be tackled by the design of ‘next generation’ CARs (Figure 4). Many of these improvements were originally applied in CAR T cells, which are more advanced in development, but are also relevant for TCR-modified T cell therapy.

#### 3.4.1. The Selection of Targetable Tumor Antigens

Thanks to their modular nature, CARs can be designed to target a variety of tumor antigens, thereby providing a readily adaptable platform to treat a variety of cancers. In principle, the optimal tumor antigen is highly and uniformly expressed on tumor cells, while it is absent on healthy tissue, however these truly tumor-specific antigens are rare. One solution is to engineer CARs that target tumor-associated glycopeptide antigens such as Muc1 and Muc16, which stem from mutations that cause aberrant glycosylation [121]. Alternatively, naturally occurring binding domains or ligands can be used as an alternative to scFv to initiate T cell activation. For example, mutated IL-13 can be used to target the IL-13R α2 chain that is overexpressed by multiple solid tumors, and less in healthy tissue [122].

Tumors downregulate or lose antigen expression through antigen escape, thereby evading antitumor detection. Antigen escape is described in up to 30% of the patients with B-ALL treated with CD19 CAR T cells and is a major cause of treatment failure. Disruption of CD19 expression due to truncations, instead of the selection of a CD19-negative clone has been described as an underlying pathogenic mechanism [123].

**Current strategies focused on overcoming antigen escape** include the use of CAR T cells able to recognize more than one antigen present on the tumor cell surface. The use of **multi-antigen targeting CAR T** **cell** (Figure 4A) products is an attractive approach and can be obtained by mixing single-targeted cells against different antigens prior to infusion (coadministration) or by simultaneously transducing T cells with different CAR constructs (co-transduction). Various types of co-transduced CARs can be discerned: dual or trivalent CAR T cells comprise of two or three CARs in a single CAR T cell, recognizing different TAAs. **Tandem CAR T cells** are composed of two distinct scFv domains that are linked in tandem in a single CAR, in order to target two different TAAs (Figure 4B). Multi-antigen targeting CAR T cells can have diverse Boolean logic-gate operations such as ‘AND’, ‘OR’, and ‘NOT’, depending on the signal transduction pattern that is built-in [124]. Multiple preclinical studies are evaluating tandem CAR T cells with the OR-gate logic, for example CD19/CD22, which is active when at least one antigen is recognized, and shows synergistic antitumor activity when both antigens are simultaneously recognized [125]. However, these OR-gate CAR T cells are unfortunately associated with increased on-target, off-tumor toxicity, as compared to AND- and NOT-gate CAR T cells, as discussed further.

Another elegant solution to this problem is the engineering of cells able to engage full antitumor potential upon administration of **switchable adaptors** that act as bridges between tumor cells and CAR T cells. These low molecular weight adaptors contain residues able to recognize molecules expressed by cancer cells, such as folate receptors, to engage the CAR-inducing cell activation. As a single adaptor CAR can activate engineered T cells against any type of target antigen, antigen-negative relapse can be circumvented. This strategy also allows for the manufacturing of universal CAR T cells directed against a variety of TAAs, thereby rendering the development of new CARs less costly and cumbersome. Furthermore, immune-related toxicities can be controlled by regulating the administration of the bispecific adaptor. Three main categories of adaptor CARs can be discerned: tag-specific adaptor CARs, bispecific antibody-binding adaptor CARs, and constant fragment (Fc)-binding adaptor CARs, which were reviewed elsewhere [126].

As an example, **T cells specific to the Fcγ receptor** (Figure 4C) are directed to tumor antigens by the administration of antibodies. To this end, a high-affinity CD16 variant CAR was designed that can capture a tumor-specific, antigen-binding antibody through the recognition of the Ig Fc region. This approach is ‘universal’ in that the tumor specificity can be tuned by using other antibodies, and the amount of antibody that is administered can be titrated to control toxicity. Moreover, the simultaneous administration of antibodies with different specificities allows to redirect the CAR T cells toward a range of different antigens, which is relevant when targeting tumors with highly heterogeneous antigens [127]. The use of adaptor CAR T cells is aimed at combining the benefits of BiTE molecules (discussed above) with the power of ex vivo-activated CAR T cells, and both strategies were compared thoroughly in a recent point–counterpoint discussion [128,129].

Moreover, in order to be able to target multiple antigens simultaneously, CAR T cells can also be engineered to secrete **BiTEs** to engage and enhance the endogenous immune system. Recently, it was reported that CAR T cells able to secrete BiTEs can overcome antigen escape in preclinical models of B-ALL and circumvent antigen heterogeneity in a solid model of glioblastoma [130].

It is important to note that any strategy applied to broaden tumor antigen recognition entails the risk of simultaneously increasing **on-target, off-tumor toxicity**. Therefore, multiple approaches have been set-up to increase the specificity toward antigens that are associated with, but not exclusively expressed on, tumor cells. In particular, Boolean AND and NOT gate designs are implemented to create multi-input receptors that activate T cells only when the specific combination of antigens is present. To this end, a second-generation CAR can be split-up into a first-generation CAR lacking the costimulatory domain, that has to be paired with a second, chimeric costimulatory receptor that is composed of a scFv antigen recognition domain fused to one or more costimulatory domains lacking the CD3ζ chain. The antigen for each receptor must be present to trigger robust signaling through this ‘**conditional’ CAR**, thereby yielding Boolean AND-gate logic (Figure 4D) [131].

Alternatively, AND-gate recognition could be based on a **synthetic Notch** (synNotch) receptor which, upon activation by recognition of one antigen, mediates the proteolysis and release of a transcription regulation factor that initiates the transcription of a CAR against a second antigen in a sequential way (Figure 4E) [132,133].

Next to the AND-gate logic described above, targeting specificity can be further increased by triggering CAR T cell activation in the presence of a TAA, though only when another antigen expressed by healthy cells is absent, referred to as **AND-NOT-logic**. The split, universal and programmable (SUPRA) CAR system was designed to this end in which the CAR expresses a leucine-zipper ectodomain instead of a ligand-binding domain [134]. This CAR has to be reconstituted with an exogenous scFv fused to a matching leucine zipper to enable T cell activation in the presence of a TAA (ZipFv). A second ZipFv can then be designed and administered, which recognizes a self-antigen and competes against the ZipCAR to circumvent the assembly of a functional CAR when self-antigens are present (Figure 4F). In general, any strategy that relies on the in trans presence of a soluble biopharmaceutical component will be complicated by the pharmacokinetic and tissue distribution factors of these molecules.

NOT-gate CAR T cells, also referred to as **inhibitory CARs** (iCARs), are an alternative approach to tackle on-target, off-tumor toxicity. These iCARs recognize antigens that are expressed on normal tissue and absent on tumor tissue, and are coupled to the signaling domain of an inhibitory receptor such as PD-1 or CTLA-4 (Figure 4G) [135]. This CAR is co-expressed with the CAR targeting the antigen of interest and prevents autoreactivity to bystander tissues. As the initial effect of the iCAR is temporary, the T cells can still function upon a subsequent encounter with the antigen recognized by their activating receptor.

Although these Boolean gating strategy increases target antigen specificity and reduces the risk of on-target, off-tumor toxicity, they also come with some limitations. For example, as the absence of either one of the target antigens is sufficient to allow tumor cells to avoid detection, the risk for tumor escape is increased. Tumor specificity can also be increased by equipping CAR T cells with COVERT molecules (Cytoplasmic oncoprotein verifier and response trigger), which are granzyme B molecules that are fused to an N-terminal inhibitory peptide sequence. Once the tumor is reached, this inhibitory peptide is proteolytically removed by tumor-associated intracellular proteases to render granzyme B active [136].

#### 3.4.2. Homing of CAR T Cells to the TME

ACT for solid tumors is particularly challenging because of the additional need to penetrate and endure the adverse TME, a hurdle which is absent in the treatment of hematological malignancies. The trafficking of T cells to their targets depends on sensing chemokines. As most tumors exhibit an altered chemokine milieu and some adhesion factors are lost on tumor endothelia, T cell penetration and migration is impaired. Furthermore, several chemokine receptors are downregulated on the T cell surface upon extensive ex vivo propagation.

Strategies are emerging to improve the homing of effector T cells through introduction of **chemokine receptor encoding genes** that match the chemokines in the TME [137]. For example, CAR T cells are engineered to co-express the chemokine receptor CXCR2, which binds to several ligands (CXCL1, 2 and 5) that show a relatively high expression level in hepatocellular carcinoma (Figure 4H) [138].

The tumor stroma consists of fibroblasts, immune cells, extracellular matrix and vasculature that constitute a hostile TME. Fibroblast activation factor (FAP) is expressed on CAFs and has been targeted in an attempt to improve CAR T cell infiltration [139]. T cell penetration can also be enhanced by transgenic expression of enzymes such as **heparanase** to degrade the physical barriers of the extracellular matrix (Figure 4I) [140].

In order to entirely circumvent the hurdle of suboptimal T cell homing, CAR T cells can be locally administered, which has already been explored in patients with solid tumors, but with varying success rates. In a recent study, patients with metastatic breast cancer were treated by the intratumoral administration of mRNA encoding an anti-c-Met CAR, and an antitumor effect with low toxicity was observed [141]. Alternatively, intracranial, intrapleural, intrahepatic, intraperitoneal, and intratumoral administration of CAR T cells showed better antitumor activity and reduced toxicity in preclinical studies [142,143,144]. As a complementary approach, CAR T cells can be embedded in functionalized biopolymer scaffolds, which promote robust T cell proliferation and release cells in a sustained manner when implanted at the site of tumor resection [145].

#### 3.4.3. CAR T Cell Persistence and Fitness in the TME

In the clinical trials reported so far, patients receive T cell products comprising a combination of CD4^+^ and CD8^+^ naive and memory T cells generated by mixed propagation from a patient’s total CD3^+^ T cell population, implicating that each patient receives a therapeutic agent with variable cellular composition. This variation might influence the efficacy of T cell therapy and complicates the comparison of outcomes over different products, patients, and across trials. Recently, it was postulated that CAR T cell products composed of defined CD8^+^ and CD4^+^ T cell subset ratios can result in uniform potency [146].

The maturation stage of amplified T cells substantially impacts the redirected antitumor activity and CAR T cell persistence. Although effector memory T cells have strong cytotoxic characteristics, only central memory T cells and less differentiated T cell subsets (naive and stem-cell-like memory T cells) are proficient for in vivo expansion, survival, and long-term persistence. CD62L^+^ enriched CAR T cells are currently explored in clinical trials [147]. Although these T cell subsets are of low abundance in peripheral blood, optimized cell culture conditions might improve their enrichment. However, it is still unresolved as to how to keep CAR T cells in the early stages of maturation, in particular after repetitive CAR activation after transfer to the patient. The fact that many ACT contestants are heavily pretreated, advanced cancer patients with blunted T cell function has led to efforts to use alternative cell sources for ACT, as is described further.

CAR T cell production and culturing in the presence of IL-7 and IL-15 instead of the previous default use of IL-2 enables the preservation of earlier differentiation, mediating superior antitumor effects [148]. Furthermore, genetic engineering leading to the over-expression of Jun, which, in combination with Fos, forms the transcription factor AP-1, can also augment expansion and extend the survival of adoptively transferred T cells after reinfusion, by driving the transcription of IL-2 [149].

**Armored CAR T cells, or ‘T cells redirected for antigen-unrestricted cytokine-initiated killing’ (TRUCKs)**, are fourth-generation CAR T cells engineered to constitutively or inducibly secrete cytokines that enhance proliferation and effector function (Figure 4J). IL-12 was one of the first cytokines to be constitutively expressed together with the CAR in order to evade immunosuppression in the TME [150]. However, concerns emerged over increased toxicity upon constitutive cytokine production. This led to the engineering of CAR T cells with inducible IL-12 expression by driving its transcription under the inducible NFAT promoter. In this way, IL-12 production is dependent on CAR T cell signaling and the cytokine remains localized to the TME [151]. IL-12 TRUCKs can induce a secondary immune response by attracting and activating M1 type macrophages into the tumor site that eliminate cancer cells that are possibly invisible to CAR T cells due to CAR-targeted antigen absence [151].

Furthermore, armoring CAR T cells with IL-15 expression can augment effector function and enhance fitness. IL-15 maintains a naive and central memory phenotype with increased expression of anti-apoptotic proteins and reduced PD-1 expression [152]. Many other cytokines including IL-7, IL-18, IL-21, and IL-23 have now been tested for their ability to enhance CAR T cell antitumor responses and an overview can be consulted in the recent review of Glover and colleagues [153].

In addition to the expression of cytokines, CAR T cell expansion and function can be improved by the introduction of **cytokine receptors** in these cells (Figure 4K). For example, a constitutively active IL-7 receptor enhances CAR T cell expansion, survival and antitumor response by restoring responsiveness to IL-7 and promoting Th1 responses without simultaneously stimulating Tregs, which is a disadvantage of many other cytokine-producing CAR T strategies [154]. However, generating transgenic cells with a constitutively active growth-promoting receptor is likely to raise regulatory safety concerns with regard to potential oncogenic transformations.

Currently, new approaches are being evaluated to design CARs with alternative co-stimulatory domains or to provide costimulation with a second molecule expressed in the CAR T cells [155]. As an example, the constitutive expression of costimulatory ligands on the CAR T cell surface can potentiate T cell activation and persistence by both auto- and trans-costimulation. Several ligands from the Ig superfamily and the TNFR superfamily such as CD80, CD86, 4-1BBL, and OX40L have been shown to enhance T cell proliferation and cytokine production upon antigen engagement and the combination of two of these ligands; in particular, CD80 and 4-1BBL result in sustained T cell expansion in vivo and the rejection of established tumors [156,157].

Furthermore, CAR T cell costimulation can be triggered by a drug. iMC, a molecule consisting of two dimerizing domains and costimulatory domains derived from MyD88 and CD40, can be activated by a chemical inducer of dimerization (CID) (**Split CAR** in Figure 4L). As costimulation is only activated in the presence of CID, a layer of safety is built in to limit T cell activation. In this way, HER2.iMC CAR T cells demonstrate enhanced survival and effector function at a lower dose [158].

One of the major problems that still remains is the functional deterioration of effector T cells in the TME due to chronic activation in the tumor, which leads to T cell exhaustion. One potential strategy is the combination of CAR T cell therapy with ICI, as described above. Nivolumab, Ipilimumab, and Pembrolizumab are currently being explored as adjuvant in CAR T cell clinical trials [159]. Furthermore, multiplexed CRISPR-based gene editing methods allow to knock out the inhibitory receptors in CAR T cells and are currently under clinical investigation [75].

An alternative approach to augment the antitumor immunity in the suppressive TME is to engineer T cells with synthetic receptors that **rewire or inhibit the downstream signaling pathways of immunosuppressive soluble factors** such as TGF-β (Figure 4M). For example, co-expression of a TGF-β dominant negative receptor (DNR) lacking a signaling motif competes with the inhibitory cytokine and leads to increased antitumor immunity, as shown in a melanoma model [160]. Additionally, synthetic inverted cytokine receptors were engineered that target other suppressive cytokines, including IL-4. A chimeric IL-4 receptor was designed in which the extracellular domain of the IL-4 receptor is fused to the intracellular domain of the IL-2 receptor beta chain or the IL-7 receptor, thereby enhancing both T cell proliferation and tumor cell killing upon co-expression with a CAR [161]. Likewise, a CAR that targets PD-L1 through an extracellular PD-1 domain coupled to co-stimulation through CD28 converts the inhibitory into an activating signal. This PD-1.CD28 **switch CAR** thus outcompetes PD-L1-induced inhibitory signaling (Figure 4N) [162].

Therapeutic vaccination is a well-established approach to enhance endogenous T cell responses against cancer [163,164]. The first proof-of-concept that efficacy of CAR T cells can be boosted by vaccination dates from 2018, when Akahori and colleagues demonstrated a CAR T cell targeting WT1 antigen peptide presented by MHC. Vaccination of CAR T cell-treated mice with activated DCs pulsed with WT1 peptide, enhanced CAR T cell expansion in the blood and increased infiltration and activation within tumors, leading to increased antitumor immunity [165]. However, due to the sophisticated and expensive manufacturing process and the dependency on MHC, DC-based vaccinations are not an ideal strategy to enhance CAR T cell efficacy in vivo. Alternative approaches include the engineering of CAR T cells from virus-specific endogenous T cells or the introduction of a CAR together with a second antigen receptor specific for a target peptide and the subsequent vaccination of the patient with the viral/secondary antigen to boost CAR T cell therapy [166]. However, all of these vaccination approaches suffer from being MHC restricted.

Recently, Darrell Irvine’s group presented a synthetic amphiphilic CAR ligand that is delivered in vivo selectively to lymph node APCs, where it decorates the surface of APCs and engages CAR T cells in an MHC-independent way [167].

Furthermore, BioNTech Corp. recently developed a nanoparticulate RNA vaccine approach termed ‘CARVac’ that is fully compatible with conventional CAR T cell therapy [168]. The vaccine comprises of an intravenously administered liposomal antigen-encoding RNA and delivers RNA into the lymphoid compartment, where it leads to selective uptake and the expression of the native antigen on the surface of DCs. An advantage of CARVac is that single-stranded RNA as a natural Toll-like receptor ligand combines the delivery of the antigen with adjuvant activity in one molecule, resulting in lymph node DCs expressing high levels of antigen and co-stimulatory molecules. This ultimately leads to robust CAR T cell stimulation in vivo and the increased expression of memory markers, improved persistence, and antitumor efficacy.

#### 3.4.4. CAR-Related Toxicities

Severe toxicities are a major concern associated with T cell engaging therapies and often implicate intensive care hospitalization. Other than the on-target, off-tumor toxicity described above, cytokine release syndrome (CRS) and immune effector cell associated neurotoxicity syndrome (ICANS) are often observed [169].

CRS is caused by the release of a large number of cytokines leading to systemic inflammation and is observed in up to 80% of patients that were administered anti-CD19 CAR T cells. The clinical manifestations of CRS include high fever, skin rash, vomiting, and nausea. These symptoms are generally caused by abnormal activation of effector T cells and the subsequent release of high levels of IFN-γ and other cytokines, which in turn can instruct macrophages to release high levels of IL-6 and IL-10. IL-6 is the key driver in this pathological process, as well as other cytokines, including TNF, IL-2, GM-CSF and IL-5. Severe CRS can be treated by the administration of dexamethasone and tocilizumab, an anti-IL-6 receptor mAb.

Neurological adverse effects or ICANs can be attributed to the redistribution of activated T cells. Activated T cells are hypothesized to interact with and disrupt the blood–brain barrier, which leads to local inflammation in the central nervous system and symptoms as aphasia, altered level of consciousness, impaired cognitive function, motor weakness, seizures and cerebral edema [170]. Unfavorable neurological events can occur either during CRS or after its resolution. In conclusion, there is a critical need to minimize undesirable immune responses.

One strategy to mitigate CRS is to genetically delete cytokines associated with toxicities, or to induce the secretion of molecules that inhibit their biological action, such as soluble cytokine receptors. The deletion of the GM-CSF encoding gene specifically in CAR T cells, or its neutralization through the administration of Lenzilumab, improved the antitumor activity [171]. Similarly, the activity of IL-1 secreted by macrophages can be counteracted by engineering of CAR T cells to constitutively express the IL-1 receptor antagonist (IL1RN), or by administration of anakinra, a soluble form of IL-1Ra [172]. Furthermore, in order to enhance the safety of CAR T cell therapy, so-called ‘smart’ T cells, which are equipped with a suicide gene or include synthetic control devices, are under preclinical and clinical investigation and are described in more detail below [173,174].

Several existing or emerging methods are currently evaluated to limit the toxicity of CAR T therapy. The administration of an external molecule (either a monoclonal antibody or a chemical compound) can then act as an antidote to shut down overactivated CAR T cells.

At the moment, two **suicide genes** have been evaluated in clinical trials: the gene encoding herpes simplex virus thymidine kinase (HSV-TK) and the gene encoding inducible caspase-9 (iCasp9) (Figure 4O). HSV-TK is the most extensively studied suicide gene in humans and is described to be effective and safe [175]. When Ganciclovir is administered, a toxic molecule is formed and apoptosis is initiated. However, due to immunogenicity of the HSV-TK, the slow onset of response, and the requirement for cell proliferation, attention has now shifted toward the inducible caspase 9 system [176]. This iCasp9 system dimerizes rapidly upon administration of an inert small molecule AP1903, which leads to its activation and the induction of CAR T cell apoptosis [177]. Following compelling preclinical results, phase I clinical trials evaluating the safety and efficacy of the iCasp9 CAR T technology were initiated for multiple indications. A downside of the use of suicide genes is the corresponding decrease in antitumor activity and, therefore, these strategies are only used in case of life-threatening toxicities.

Alternatively, mAbs approved for clinical use can be used to eliminate CAR T cells through the recognition of co-expressed, truncated cell surface proteins, also referred to as **elimination markers** (Figure 4P). In this way, CAR T cells are depleted through ADCC and complement cytotoxicity. CD20 (and use of the anti-CD20 mAb rituximab) and the truncated human EGFR (huEGFRt recognized by the mAb cetuximab) are two of the most commonly used elimination markers [178,179]. As rituximab will not only eliminate the elimination marker-expressing CAR T cells, but also both malignant and normal CD20-expressing B cells, this approach has not reached clinical trials yet. However, the huEGFRt-cetuximab switch led to promising preclinical results and is now being evaluated in multiple phase I clinical trials.

Selective expression of the CAR on the cell surface can also be obtained by engineering **a small-molecule-assisted shutoff** (SMASh) as part of the CAR sequence (Figure 4Q). In this CAR design, a self-cleaving site is controlled by a drug-sensitive viral protease paired to a degradation signal or degron that is able to induce proteolysis of the CAR protein. When in the ‘ON’ state, self-cleavage leads to proteasomal degradation of the degron and subsequent CAR expression on the cell surface. Upon administration of the inhibitor asunaprevir, the CAR is switched to the ‘OFF’ state, in which the degron sequence is retained, allowing proteasomal degradation of the CAR-degron protein within 48 h [180]. As this type of OFF-switch is not lethal, it allows CAR T cells to be reversibly controlled in a cost-effective way. Moreover, here, the immunogenicity of the exogenous protein components has to be evaluated.

Alternatively, the tyrosine kinase inhibitor Dasatinib can be administered as a pharmacological OFF-switch to disrupt signaling downstream of the activation domain CD3ζ by the inhibition of LCK. In this way, Dasatinib temporarily inactivates CAR T cells and full antitumor activity is restored upon removal of the drug [181].

As an alternative to the elimination of overactive CAR T cells that can cause life-threatening toxicities, the engineering of an activating ON-switch is an interesting approach that allows the precise control of CAR T cell timing, site of action, and dosing.

The potential advantages of the **tetracycline-ON** (Tet-ON) system were evaluated in multiple preclinical studies (Figure 4R) [182]. This inducible gene expression system is based on a reverse Tet trans-activator fusion protein (rtTA), which activates the CAR gene transcription upon administration of doxycycline, and has been tested in multiple indications such as hepatocellular carcinoma (CD147 CAR), B cell malignancies (CD19 CAR), and multiple myeloma (CD38 CAR) [183,184,185].

Another ON switch approach involves engineering CARs to recognize **switchable adaptors** (Figure 4S) that act as bridges between tumor cells and CAR T cells, as already mentioned above.

#### 3.4.5. Labor and Cost-Intensive CAR Production Process

The manufacturing of CAR T cells at a clinical scale involves complex procedures of T cell isolation, genetic engineering, and the expansion of redirected T cells ex vivo prior to their infusion back into the patient. Therefore, this is a complex and costly manufacturing process that is associated with multiple clinical hazards. Moreover, these processes require substantial technical expertise that is only available in a few specialized centers worldwide. The most crucial hurdle that is to be tackled is the development of new approaches to make this complicated and risky cell therapy safer, affordable, and customizable. In this way, CAR T cell therapy may, in the end, outcompete chemotherapy as a front-line therapy widely accessible for a larger population.

Currently, most adoptive cell therapy trials focus on the treatment of patients with autologous CAR T cells. This individualized therapy is labor- and cost-intensive and limits the widespread applicability of CAR T cells. Further, it was recently shown that patients requiring bridging therapy between the time of apheresis and CAR T cell infusion have a worse overall response, highlighting the downside of waiting for the manufacturing of autologous cell therapy products [186]. As CAR T cell therapy is presently only approved as a last-rescue line of therapy, bridging therapy is almost always necessary for patients.

The ability to use cells from healthy donors, referred to as allogeneic CAR T cells, could potentially address these issues. These allogeneic CAR T cells are potentially ‘universal’ T cells that can be manufactured in advance and applied ‘off-the-shelf’ to a number of patients. However, these allogeneic approaches are confronted with some major challenges: upon administration, allogeneic T cells can cause life-threatening graft-versus-host disease (GvHD) due to HLA mismatching, or may be rapidly rejected by the host immune system, thereby limiting their antitumor potential.

Alloreactive αβ T cells that react with foreign MHC molecules are the key mediators of both transplant rejection and GvHD, and several strategies have been designed to administer allogeneic CAR T cells with a reduced risk of GvHD [187]. First of all, the **T cell source** used to engineer allogeneic CAR T cells can be optimized. Currently, T cells are mainly derived from PBMCs and, in limited cases, from umbilical cord blood. In this way, there is an opportunity to generate a biobank, containing cells expressing different HLA subtypes, which allows for the selection of batches that match the HLA type of the patient. Furthermore, the use of umbilical cord blood-derived CAR T cells could be associated with reduced risk and severity of GvHD due to their antigen-naive status and impaired NFAT signaling [188]. In principle, CAR T cells may also be obtained from induced pluripotent stem cells (iPSCs) with a theoretically unlimited capacity to self-renew [189]. Furthermore, in patients who received allogeneic stem cell transplantation but experience a relapse, CAR T cells can be engineered from the original stem cell donor to strengthen the graft-versus-tumor effect while limiting the risk of GvHD [190].

Another approach consists of engineering alternative cell types to express a CAR, thereby avoiding the use of αβ T cells. Cell types that are suitable for CAR T cell therapy are characterized by a cytotoxic capacity that can be redirected toward the tumor through a cell surface receptor, and must be readily available from sources such as PBMCs and stem cells. Moreover, they should be relatively easy to manipulate and expand ex vivo. Cell types that meet these criteria include NK cells, NKT cells, and γδ T cells [187].

**NK cells** were originally identified as tumor-killing cells and are integral to natural tumor immunosurveillance. NK cells kill their targets in an antigen-independent manner without causing GvHD, as they are highly cytolytic toward cells that display the cognate ligands, which, upon ligation, lead to an appropriate balance of NK signaling through activating receptors (for example, CD16 and NKG2D) versus inhibitory receptors (for example, receptors binding MHC class I molecules marking ‘self’). Tumor cells in their turn have evolved mechanisms to evade NK cell killing. Therefore, the engineering of NK cells with a CAR to reinforce their antitumor potential is an attractive strategy [191]. As NK cells are present at a relatively low concentration in peripheral blood, strategies were developed to specifically enrich and expand them ex vivo. For example, NK cells derived from umbilical cord blood engineered to express IL-15, together with a CAR targeting CD19, showed prolonged persistence and activity in preclinical studies [192]. Furthermore, the use of the NK92 cell line has been approved for use in humans and constitutes a renewable source to produce CAR NK cells [193]. Additional information on preclinical and clinical studies on CAR NK cells was recently reviewed by Gong and colleagues [194].

**NK T cells**, on the other hand, are a subset of T lymphocytes that express NK cell surface markers. A subset of NK T cells, invariant NK T cells (iNKT cells), express a highly restricted TCR that recognizes specific lipid antigens presented by the non-polymorphic HLA class I-like molecule CD1d expressed on B cells, APCs, and some epithelia [195]. Because of their peculiar TCR constitution and antigen recognition modality, iNKT cells do not display any GvHD induction potential. This makes them an interesting cell population for off-the-shelf development. When iNKT cells are engineered with a CD19 CAR, they show strong anti-lymphoma properties by targeting both CD19 and CD1d expressed on lymphoma cells [196]. As this cell type is limited in number (<0.1% of circulating T cells), efficient massive ex vivo expansion protocols are required.

Another group of immune effector cells that is being explored as potential platform for CAR engineering are γδ T cells. These cells comprise a relatively small subset of T cells (between 1 and 10% of our CD3^+^ T cells) and typically recognize their stress-induced ligands independent of antigen processing and MHC/HLA restriction and therefore do not cause GvHD. This T cell subtype is considered to act at the crossroad of the innate and adaptive immune system, as they rapidly respond to their TCR triggering, while they also frequently co-express functional receptors of innate immune cells, such as activating NK receptors (e.g., NKG2D) [197]. Furthermore, these cells have the capacity to differentiate into professional antigen presenting cells upon activation. γδ T cells can be expanded to large numbers ex vivo by use of zoledronate, concanavalin A, or artificial APCs. When they are redirected with a CAR-targeting disialoganglioside GD2 which is overexpressed in gliomas, their cytotoxicity is enhanced [198]. Similarly, CD19-targeting γδ CAR T cells demonstrated antitumor activity both in vitro and in vivo [199]. Further challenges, including improving in vivo persistence, are to be addressed prior to clinical application.

Next to the optimization of the T cell donor, **gene editing approaches** are being developed to obtain deletion of the TCR and HLA class molecules in αβ T cells. The gene encoding for the TCR constant α chain (TRAC) is the most straightforward gene to disrupt to prevent the expression of a functional TCR. Additionally, deletion of CD52 in donor T cells is performed, making them resistant to Alemtuzumab, an anti-CD52 monoclonal antibody that can be used to eliminate the host T cells to prevent allo-rejection [200]. More recently, a CRISPR Cas9-based strategy combined with AAV delivery was developed to incorporate the CAR construct into the TRAC locus. Because of this targeted integration approach, some advantages arise, including reduced insertional mutagenesis and physiological CAR expression under the control of the endogenous TCR promoter. Because HLA class I molecules are key mediators of immune rejection of the transplanted CAR T cells, a complementary approach is to delete MHC class I molecules such as β2-microglobulin in allogeneic CAR T cells [201].

Recently, an automated, GMP-compliant and clinical-scale T cell engineering process was described to produce off-the-shelf CAR T cells on an extended CliniMACS prodigy platform containing an in-line electroporation unit. This set-up allows for the combined lentiviral delivery of a CD19 CAR construct with the transfer of mRNA encoding a genetic engineering cassette to disrupt the TRAC locus. In this way, a CAR T cell product is generated that is more than 99.5% free of the endogenous TCR, with high quality and with conserved cytotoxic potency [202].

Even if efficient allogeneic T cell production can be achieved, CAR T cells will still remain a complex product to manufacture. The ultimate goal would be to transfer the CAR genes directly and selectively to T cells in the patients in situ. The research group of Buccholz has performed the first step toward in vivo reprogramming of CAR T cells using lentiviral vectors (LVs) that use the CD8 α chain as an entry receptor (CD8-LV). A single systemic injection of CD19 CAR encoding CD8-LVs into immune-deficient mice engrafted with human blood cells generated in vivo CAR T cells, which effectively eliminated human B cells and CD19^+^ tumor cell lines [203,204]. It is important to test the consequences of delivering the CAR not only into CD8^+^, but also into CD4^+^ T cells to further boost the antitumor activity. The tools for simultaneous specific in vivo generation of CD4^+^ CAR T cells are also available [205].

Very recently, proof-of-principle was obtained for an adeno-associated virus delivering CAR gene therapy (ACG) [206]. AAV has become an essential therapeutic gene delivery vector in recent years and is frequently used in clinical applications in academia and industry [207]. Nawaz and colleagues have now designed an AAV vector encoding a CD4 targeting CAR that successfully led to high CAR T cell numbers in vivo. Furthermore, the CAR T cells were shown to cause effective tumor regression in a T cell tumor model. However, further studies are required to design an ACG with ligand coupling or other modifications in the AAV capsid to enable cell-specific delivery of the CAR gene [208].

Over the last years, a variety of nanocarrier systems has been evaluated, as they comprise an appealing vehicle for highly targeted therapy. Inexpensive DNA nanocarriers were designed that can quickly and specifically introduce CD19-targeting CAR genes into T cells that are circulating within the patient. These nanoparticle reprogrammed T cells continued to express their receptor for weeks, allowing them to serially kill tumor cells and differentiate into memory T cells [209].

The question of whether in vivo generated CAR T cells could be as effective as ex vivo generated CAR T cells remains to be investigated in more detail. Furthermore, standardization of the vector particle dosing will be a particular challenge. Beyond that, further preclinical testing, especially in large animal models, will be required to move this approach further toward translation. Nonetheless, this approach might, in the end, lead to a single injection of a vectorized medicine into the blood stream, thereby circumventing the current cost-intensive ex vivo production of CAR T cells, making this therapy more broadly available to patients. Moreover, the increased flexibility associated with this approach would facilitate the development of novel immunotherapeutic concepts, including indications beyond cancer, such as autoimmune diseases and infectious disease.

### 3.5. Applications of CAR T Cell Therapy for Indications beyond Cancer

Based on the knowledge obtained from CAR T cell engineering in cancer research, many efforts are being made to develop similar therapies for patients affected by autoimmune diseases, allergies, chronic viral, and acute invasive fungal infections [210]. Despite differences in etiology, common features in pathogenesis of these diseases allow them to be treated with CAR T cells. These include disease-specific cellular components that mark cells as infected, over-activated, or over-expanded. Additionally, CAR T cells can act as a powerful substitute to the human immune system that is (partly) dysfunctional because of the disease.

#### 3.5.1. CAR T Cells to Treat Autoimmune Diseases

**Figure 5:**
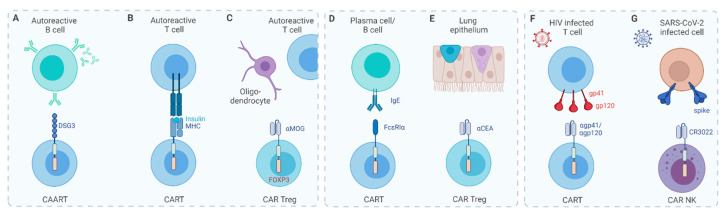
Applications of CAR T cell therapy beyond oncology: DSG3: Desmoglein 3; CAART: chimeric autoantibody receptor T cell; MOG: myelin oligodendrocyte glycoprotein. (**A**) Autoreactive B cell, (**B**) Autoreactive T cell, (**C**) Autoreactive T cell, (**D**) Plasma cell/B cell, (**E**) Lung epithelium, (**F**) HIV infected T cell, (**G**) SARS-CoV-2 infected cell.

Autoimmune diseases are generally characterized by a failure of T cell tolerance toward self-antigens and anti-autoimmune CAR T cells are designed with the aim of eliminating these autoreactive clones of immune cells. In a first strategy, chimeric autoantibody receptor T cells (CAAR), also referred to as B cell antibody-targeting receptor T cells, function to target autoreactive B cells, carrying receptors to the specific autoantigen. This leads to the direct removal of surface Ig memory B cells, and to the indirect elimination of plasma cells that produce the autoantibodies causing the disease. As an example, CAAR-T cells were designed to treat the autoimmune disease pemphigus vulgaris, characterized by skin blisters and mucosal membranes, and mediated by autoantibodies targeting keratinocyte adhesion protein desmoglein (Dsg)3, as shown in Figure 5A. CAAR-T cells carrying the Dsg3 autoantigen as their extracellular domain showed high efficacy in the elimination of anti-Dsg3 B cells, and a first clinical trial is currently ongoing [211].

Similarly, CAR T cells can be used to target auto-antigen-presenting APCs or the T cells responding to such APCs. In this regard, multiple studies have been focusing on Type 1 diabetes, an autoimmune disease mediated by both CD4^+^ and CD8^+^ T cells, leading to the destruction of insulin-producing islet β-cells. A CAR construct with a peptide-bound MHC complex coupled to CD3ζ signaling motif targets CD8^+^ T cell clones carrying a TCR specific to for example an insulin-B chain peptide (Figure 5B) [212].

As Tregs are usually suppressed in autoimmune diseases, another interesting concept aims to restore immune tolerance through the induction of a regulatory T cell phenotype. CAR Tregs are CAR T cells converted to Tregs by the introduction of FOXP3. CAR Tregs are then instructed to recognize and regulate autoimmune T cells through induction of anergy and clonal deletion. This idea was evaluated as a potential treatment for multiple sclerosis, in which pathogenesis is driven by autoreactive T cells recognizing myelin epitopes. CAR Tregs were designed from CD4^+^ T cells to target myelin oligodendrocyte glycoprotein in order to bring Tregs in close proximity to oligodendrocytes to avoid local immune outbreaks (Figure 5C) [213].

Additionally, three clinical trials, which are being currently conducted, are focused on the administration of CAR T cells against autoimmune diseases by depleting the complete B cell population instead of only the autoreactive clones. Anti-CD19, -CD20, and -BCMA CARs are studied here for the treatment of generalized myasthenia gravis, systemic lupus erythematosus, and neuromyelitis optica (NTC04146501, NTC03030976, and NTC03605238, respectively). While this approach may appear very drastic, anti-CD20 antibody therapy has successfully been used to treat autoimmune disease, and these CAR T therapies are inspired by the finding that side-effects are tolerable with appropriate dosage.

#### 3.5.2. CAR T Cells to Treat Allergies

Allergies are dominated by a Th2-driven immune response that can potentially be modulated and controlled by the function of Tregs. Furthermore, IgE class antibodies produced by B cells play an essential role in the pathogenesis of allergic diseases. Binding of IgE to its high-affinity receptor FcεRI on mast cells, eosinophils, and basophils causes degranulation and the release of inflammatory mediators. In this context, CAR T cells were designed that include the extracellular domain of the FcεRI α-chain that recognize the transmembrane form of IgE on germinal center B cells, plasma cells, and memory B cells (Figure 5D) [214].

Allergic asthma is characterized by low numbers and immunosuppressive activity of Tregs combined with excessive Th2 driven responses leading to airway inflammation. Tregs were redirected to the lungs by engineering a CAR that recognizes CEA, a glycoprotein presented on the surface of lung epithelium, and which led to promising pre-clinical results (Figure 5E) [215].

#### 3.5.3. CAR T Cells to Treat Infectious Diseases

Several CAR T cell therapies targeting infectious diseases have been described and were recently reviewed [216]. In particular, tremendous progress has been made in anti-HIV CAR T cell therapy, which already reached clinical trials. Combinatorial antiretroviral therapy can effectively suppress HIV-1 replication but complete eradication of the latent reservoir of infected cells is still challenging. As CD8^+^ T cell cytotoxicity against HIV-1 infected cells plays an important role in the management of infection, the application of CAR T cells for HIV treatment provides a promising new therapeutic avenue [217]. The main target for anti-HIV-1 CAR T therapy is the gp120 region of the HIV envelope glycoproteins that is expressed on the surface of infected cells. The first anti-HIV CAR was designed through the incorporation of the CD4 transmembrane and extracellular domains that target gp120 [218]. In following optimizations, anti-HIV CARs were developed based on broadly neutralizing monoclonal antibodies targeting gp120 and gp41 [219] (Figure 5F). Additional engineering to, for example, delete the CCR5 locus can protect the engineered cells themselves from HIV infection [220].

CAR T cells targeting other viruses such as HBV, HCV, CMV, and opportunistic fungal infections such as *Aspergillus fumigatus* are still in early preclinical testing. The use of CAR-transduced immune cells to target virally infected cells has very recently drawn the attention of the scientific community for the treatment of COVID-19 caused by the contagious SARS-CoV-2 virus. In this regard, a promising therapeutic strategy is focusing on the engineering of NK cells, the effector lymphocytes of our innate immune system with the primary function to destroy cancer cells and virus infected cells. NK cells are activated through either direct recognition of viral proteins or through inhibitory NK receptor signaling in the case of MHC class I downregulation on the surface of infected cells [221]. CAR-NK cells targeting the SARS-CoV-2 spike protein were developed based on the scFv domain of the antibody CR3022 (Figure 5G) [222]. Additionally, a clinical trial is currently evaluating bispecific CAR-NK cells in which the activating receptor NKG2D recognizes infected cells and the ACE2 receptor binds the SARS-CoV-2 spike protein (NTC04324996).

## 4. Conclusions

T cell-based cancer immunotherapies have emerged as a powerful tool in oncology. Nonetheless, it has taken many years of basic scientific research and subsequent translation to the clinic in order to demonstrate the power of immune system manipulation for the treatment of cancer. Further research into how T cells are regulated and their interplay with other immune cells such as DCs and NK cells will allow us to increase the strength of this approach even further. Thanks to the rapid development of genome editing technology, it is feasible to evaluate ambitious concepts, and to apply novel and often revolutionary clinical approaches. Although various challenges associated with CAR T cell therapy still remain to be solved, the overall great promise is supported by encouraging clinical results and the ongoing research in this field offers hope for the clinical application of cell therapies for cancer as routine rather than investigational strategies at the margins of frontline treatment.

## Figures and Tables

**Table 1:** Comparison of the main ACT products. Table based on Rohaan et al. 2018 [48]. TILs: tumor-infiltrating lymphocytes; TCR: T cell receptor; CAR: Chimeric antigen receptor; CRS: cytokine release syndrome; CNS: central nervous system.

	TILS	TCR T Cells	CAR T Cells
**Production Method**	T cell isolation from tumor	Leukapheresis	Leukapheresis
**T** **arget Antigen**	Both intracellular and cell surface	Both intracellular and cell surface	Cell surface
**Need for Lymphodepletion**	Yes	Yes	Yes
**Need for Supportive IL-2**	Yes	Varying	No
**Specificity**	Polyspecific	Monospecific	Monospecific
**MHC** **Restriction**	Yes	Yes	No
**Off-the-Shelf Potential**	No	Yes	Yes
**T** **oxicities**	Lymphodepletion regimen IL-2 mediated	Lymphodepletion regimen On target, off tumor CRS	Lymphodepletion regimen On target, off tumor CRS and CNS

**Table 2:** Costimulatory signaling molecules used in CAR T cell design. Table adapted from Weinkove et al. [100].

Costimulatory Molecule	Implications For Car Functionality	Recent Publications
**CD28**	Potent cytotoxic function Increased IL-2 production Increased CD4^+^ T cell expansion	[101]
**4-1BB**	CD8^+^ T cell central memory differentiation Increased CAR T cell persistence	[102]
**ICOS**	Increased Th1 and Th17 polarization Enhanced persistence	[103,104]
**OX40**	Suppression of Treg development Increased persistence in combination with CD28	[105]
**CD27**	Enhanced resistance to apoptosis Increased CAR T cell persistence and cytotoxicity	[106]
**MYD88/CD40**	Increased Th1 polarization Decreased levels of transcription factors associated with T_TE_ cell differentiation	[107,108]
